# A pilot study for genome wide association study (GWAS) in patients with juvenile idiopathic arthritis (JIA) and their parents

**DOI:** 10.1186/1546-0096-9-S1-P152

**Published:** 2011-09-14

**Authors:** Seza Ozen, Yesim Aydin Son, Yener Tuncel, Erkan Demirkaya, Yelda Bilginer, Cengizhan Acikel, Bilge Volkan Salancı, Ozgur Kasapcopur, Erbil Unsal, Nesrin Besbas, Mehmet Alikasifoglu

**Affiliations:** 1 Hacettepe University Faculty of Medicine, Department of Pediatrics, Pediatric Nephrology and Rheumatology Unit, Ankara, Turkey; 2 METU Informatics Institute, Department of Health Informatics, Ankara, Turkey; 3 METU Informatics Institute, Bioinformatics Graduate Program, Ankara, Turkey; 4 Gulhane Military Medical Academy, Pediatric Nephrology & Rheumatology Unit, Ankara, Turley; 5 Gulhane Military Medical Faculty, Department of Public Health, Division of Epidemiology, Ankara, Turkey; 6 Hacettepe University Faculty of Medicine, Department of Pediatrics Clinical Genetics Unit, Ankara, Turkey; 7 Istanbul University Cerrahpasa Medical School Department of Pediatric Rheumatology, Istanbul, Turley; 8 Dokuz Eylul University Faculty of Medicine, Department of Pediatrics, Division of Immunology- Rheumatology, Izmir, Turkey

## Background

GWAS are being performed to define susceptibility risk factors for JIA.

## Aim

We have performed a genome wide analysis in patients with JIA and both their parents as trio sets to define the basis of genetic susceptibility for JIA.

## Methods

A GWAS using Gene Chip Human Mapping 250K Nsp arrays was performed in 35 trio sets. The patients were grouped into those having oligoarticular or polyarticular JIA and those having SoJIA. Case-control and trio analysis are performed with plink toolset to determine JIA associated SNPs in both patient groups and associated SNPs are prioritized according to their statistical and biological relevance. Enriched genes and pathways within the prioritized SNP list are also analyzed.

## Results

Both case-control, trio analysis and chromosomal distributions of the associated SNPs revealed that there were marked differences between the associated SNP profiles of the two groups of JIA patients. The identification of the top associated SNPs in the two groups is in progress. Prioritization and gene enrichment analysis has been done in order to gain more insight to the JIA etiology at gene and pathway level. Associations were present with HLA and some previously defined loci published for rheumatoid arthritis and JIA patients. The other strong associations that were common in both patient groups were present for SNPs in the TLR pathway, matrix metalloproteinase, calcium pathways and S100A4.

## Conclusion

GWAS for case-control and trio analysis has shown differences between the systemic onset JIA compared to the others. The parental origin of transmission is also being assessed.

